# Breaking the silence about obstetric violence: Body mapping women’s narratives of respect, disrespect and abuse during childbirth in Bihar, India

**DOI:** 10.1186/s12884-022-04503-7

**Published:** 2022-04-14

**Authors:** Kaveri Mayra, Jane Sandall, Zoë Matthews, Sabu S. Padmadas

**Affiliations:** 1 Department of Social Sciences and Demography, University of Southampton, Southampton, UK; 2 Department of Women and Children’s Health, Kings College London, London, UK

## Abstract

**Background:**

Evidence on obstetric violence is reported globally. In India, research shows that almost every woman goes through some level of disrespect and abuse during childbirth, more so in states such as Bihar where over 70% of women give birth in hospitals.

**Objective:**

1) To understand how women experience and attach meaning to respect, disrespect and abuse during childbirth; and 2) document women’s expectations of respectful care.

**Methods:**

‘Body mapping’, an arts-based participatory method, was applied. The analysis is based on in-depth interviews with eight women who participated in the body mapping exercise at their homes in urban slums and rural villages. Analysis was guided by feminist relational discourse analysis.

**Findings:**

Women reported their experiences of birthing at home, public facilities, and private hospitals in simple terms of what they felt ‘good’ and ‘bad’. Good experiences included being spoken to nicely, respecting privacy, companion of choice, a bed to rest, timely care, lesser interventions, obtaining consent for vaginal examination and cesarean section, and better communication. Bad experiences included unconsented interventions including multiple vaginal examinations by different care providers, unanesthetized episiotomy, repairs and uterine exploration, verbal, physical, sexual abuse, extortion, detention and lack of privacy.

**Discussion:**

The body maps capturing birth experiences, created through a participatory method, accurately portray women’s respectful and disrespectful births and are useful to understand women’s experience of a sensitive issue in a patriarchal culture. An in-depth understanding of women’s choices, experiences and expectations can inform changes practices in and policies and help to develop a culture of sharing birth experiences.

**Supplementary Information:**

The online version contains supplementary material available at 10.1186/s12884-022-04503-7.

## Background

Obstetric violence is a violation of women’s fundamental human rights [1] against their entitlement to the highest attainable standards of health [2]. It is also recognized as a strong disincentive to facility-based births and threatens efforts made to reduce maternal mortality [3]. Respectful maternity care is the antidote to obstetric violence. Though it is a relatively new area of research, the issue of disrespect and abuse during childbirth has existed for much longer. Globally, the recent ‘What Women Want’ campaign asked 1.2 million women and girls from 114 countries what their one demand was for quality maternal and reproductive health care [4]. The top ranking response from 103,584 women was respectful maternity care which provides a clear imperative to tackle this issue [4]. This campaign received 350,696 responses in India, where it is also called ‘*Hamara Swasthya Hamari Awaaz’* [4]. This is of particular concern in a country which carries 12% of the global burden of maternal deaths [5] and has a poorly resourced health system. There is a growing body of literature documenting obstetric violence in India indicating that disrespectful maternity care is a widespread phenomenon across the states [5–9].

Researchers from across the world continue to explore the meaning, driving factors and measurement for respect, disrespect and abuse during childbirth, to integrate it into standards of person-centered care [10–12]. Understanding the context, and women’s perception and experience of respect, disrespect and abuse during childbirth, is essential to design policies and strengthen systems which provide respectful care [13].

While some forms of mistreatment, such as performing episiotomy without anesthesia, would be considered abusive in any country, context still plays a key role in understanding mistreatment during childbirth, as the meaning of respect, disrespect and abuse may vary across cultures and geographies [14–16]. Perceptions of respectful care could be influenced by a person’s socio demographic characteristics, as is reflected in the definitions and drivers of mistreatment. Sen et al. (2018) defines disrespect and abuse during childbirth reflecting from the intersectional angle stating that race, ethnicity, economic status, marital status, disability, gender identity and sexual orientation may increase women’s vulnerability to mistreatment [16]. Freedman and colleagues argue that mistreatment in obstetric service settings occurs at the individual, structural and policy levels [17]. This underlines the fact that not all types of mistreatment are intentional, and that insensitive care and lack of policies addressing inadequacies in the birthing environments can expose women to mistreatment.

Women may find it difficult to narrate their birth experience because they may not be accustomed to medical language and terms. They may also suffer the stigma and shame related to conversations in reference to body parts such as one’s genitals and the awareness that birth is a result of intercourse [15, 18, 19]. ‘Pain’ is the common explanation of their experience. The clinical language used by care providers could be difficult for women to understand and incorporate into their narrative. Studies have reported that women’s accounts of traumatic birth experience are often similar to narratives of rape victims [18–20]. The language used by survivors of both kinds of abuse include phrases such as being ‘stripped off’, ‘tethered’, ‘forcibly exposed’, ‘sexual organs put on display’, ‘disempowered’ and ‘feeling butchered like being a slab of meat’ [19]. Also, non-verbal gestures of communication such as touching the women during childbirth can be comforting or discomforting especially when done without consent. The pain experienced during childbirth often makes women vulnerable too. Reporting a traumatic birthing experience becomes secondary when women are expected to be grateful for surviving childbirth with a live baby [19]. Some women may relive the birth trauma every year on their child’s birthday as a ‘trauma anniversary’. Women may feel disgusted and depressed every year when they feel they are expected to celebrate the child’s birthday [18, 20].

Women may not be aware that certain actions or behaviours are not part of care, so even though they feel bad, they have no basis for thinking that childbirth can be a pleasant experience. Conversely, women may refrain from reporting traumatic childbirth, as they might think that such experiences are normal while others might be afraid of being silenced [3, 9, 15, 21]. Women as care-seekers may lack awareness of what good quality care really entails [11]. They may expect to be mistreated or subdued [22]. Furthermore, women may feel that they have no say in their care and thus opt to accept the abuse during childbirth [23]. Others may choose to give birth at health care another facility based on their previous birth experience [24], rather than reporting abuse. Literature highlights that under-reporting becomes clear when women’s self-reports are compared with birth observations [9]. Sometimes the expectations are so low that satisfaction is not a priority. An Indian study reported that women considered the availability of health care providers and health supplies such as medicines, as two key aspects of good quality care [13]. Similarly, women made suggestions for aspects of care such as reduction in the waiting time and provision of a sitting area while waiting as well as improvement of laboratory services to increase satisfaction with health care [25]. It is evident from the literature that abuse the provision of care is prevalent [16, 22, 26, 27].

Maternal health outcomes in Bihar are among the worst in India and there has been little or no progress. The goal of this paper is to understand women’s own experiences and perceptions of respect, disrespect and abuse while giving birth in Bihar, India. We address this goal by: 1) investigating how women perceive and attach meaning to respect, disrespect, and abuse during childbirth in Bihar and 2) documentation of women’s expectations of respectful care necessary to ensure a positive birthing experience.

### Study setting

Bihar is the third most populous state in India with a population of 104 million, and about 88% of the population resides in rural areas [28]. Bihar has the highest fertility rate in India (3 children per woman) [29], which adds to yearly increases in capacity needed for maternity services [28]. Bihar’s patriarchal culture is clearly reflected in the system of early marriage and preference for sons over daughters. The aversion and neglect towards daughters further explains the poor indicators of women empowerment when compared to national average and other states. The recent National Family Health Survey (NFHS-5) reports that 41% of the women surveyed were married before the age of 18 years and 11% of girls aged between 15-19 years were reportedly pregnant at the time of survey [29]. Women and girls are deprived of education and employment opportunities, which lower their position and status within the household and community. These, in turn, with caste-based disadvantages deter women from seeking healthcare [30]. The female disadvantage is also reflected in the distorted sex ratio at birth of 908 females per 1000 males [29].

When compared with other Indian states, women in Bihar face more gender-based challenges that make them vulnerable to experiencing violence in many aspects and phases of their lives. The challenges include high rates of domestic violence and intimate partner violence during pregnancy [31–33]. The prevalence of violence against women in the state is an indicator of the patriarchal culture in Bihar. The NHFS-5 reports that 40% of the women surveyed experienced spousal violence at some point in their life, 3% experienced physical violence from their husband during pregnancy. Among women surveyed who were aged between 18-29 years, 8% had experienced sexual violence before the age of 18 [29].

There has been a steep rise in births in health care facilities in Bihar (from 3.5 to 76%) in the last fifteen years, driven by policies implemented nationwide, such as the Janani Surakha Yojana (JSY) and a better performing referral system for emergency obstetric care. To sustain health care facility births, the JSY introduced an incentive system for women and Accredited Social Health Activists (ASHA) to encourage women to give birth in health care facilities [34, 35]. However, the policy on incentives has not contributed to a comparative reduction in maternal mortality ratio in the last 15 years. At the same time, there has been minimal increase in antenatal and postnatal care– now one of the lowest in the country (Table 1) [29]. The Government of India’s strategic interventions, such as LaQshya, aim to improve quality of care (QOC) in labour rooms, but have limited scope for improving respectful maternity care when compared to other guidelines such as WHO recommendations for intrapartum care, despite the focus on quality care improvement [35, 36]. Although, no systematic evaluations have been done in the country yet, the disproportionate increase in cesarean section rates in private hospitals amongst urban wealthier women, is also an indicator of inequities in health care provision between public and private, urban and rural areas [29].

**Table 1:** Birth related indicators for women in Bihar, India (%)

No.	Birth related indicators	Bihar 2005-06	Bihar 2015-16	Bihar 2019-20	India 2015-16
1	Percentage of women with four ANC visits	11.2	14.4	25.0	51.2
2	Percentage of women who gave birth in a health facility	19.9	63.8	76.0	78.9
3	Percentage of women who were assisted by a skilled care provider during childbirth	29.3	70.0	79.0	81.4
4	Percentage of institutional births in a public facility	3.5	47.6	57.0	52.1
5	Percentage of births by cesarean section	3.1	6.2	10.0	17.2
6	Percentage of births by cesarean section of those in public hospitals	7.6	2.6	4.0	40.9
7	Percentage of births by cesarean section of those in private hospitals	17.2	31.0	40.0	11.9
8	Percentage of women who were visited by a care provider for a check up within two days of birth	NA	10.8	59.3	62.4

Source: National Family Health Survey (NFHS), India, various years

There are studies reporting mistreatment of women during childbirth in the neighbouring state of Uttar Pradesh which has a similar sociocultural context to Bihar [7–9]. There is a lack of evidence and understanding of dignity and respect in maternal health care in Bihar, particularly in a context driven by rapid shift from home to institutional births. Addressing this research gap is key to facilitate care that is women-centered and design policies that enable provision of respectful care guided by women for a positive birthing experience.

## Methods

This is a qualitative study undertaken in Bihar, where we conducted in-depth interviews aided by a participatory visual arts-based research method called body mapping. Feminist critical theory informed all aspects of this study, which enabled an understanding of childbirth as a human experience embodied in previous birth experience, inter-subjective, contingent, and woven into personal and cultural webs of signification.

### Birthing body maps

Body mapping is a unique participatory approach that combines visual arts and therapeutic practice to guide women in the artful communication about their embodied life experiences in ways that are safe and supportive [37, 38]. Birthing maps is a term we have coined to refer to our own adaptation of the established body mapping method whereby we utilised it to explore women’s experiences of giving birth. This was an ideal method for data collection as it reduced literacy and power-related barriers to women’s ability to communicate their experiences. Collecting narratives of birth involves talking about body parts that are shameful to talk about in the local context for the women in the study. Birth mapping is a culturally appropriate method because it is flexible for adaptation and enables women to bypass stigma and share rich accounts of their experiences. The exercise started with the interviewer requesting women to lie down on the sheet in the position they gave birth in, and then drawing a life-size outline around the woman. The interviewer illustrated this by lying down on the sheet herself, which overcame hesitation from women in doing the same. Talking about birth is sensitive and body mapping helps women open up over the course of a few interactions that allows development of trust between the interviewer and interviewee. Birthing body maps have three components: 1) the birthing map, 2) the birthing story, and 3) the body key. These were co-created by the women and the researchers. The languages used on the map are the ones that women understood. For example, Fig. 2 is WR08’s birthing map where some quotes are written in English because WR08 understands both Hindi and English. The script in all the other birthing body maps are in *Hindi* only.

### Data collection

Our research was informed by a scoping study conducted in January 2019 in a rural village and an urban slum in Patna. This scoping study helped in assessing the feasibility of conducting body maps and to develop a semi-structured guide for data collection. Data were collected in November- December 2019.

This study was conducted in the Patna and Muzaffarpur districts in Bihar. Patna, as the state capital, has specialised tertiary level health care facilities accessible to urban slum dwellers. We selected the district for data collection based on the high rates of institutional births (79-87%) that exist in urban Patna. Muzaffarpur has a slightly lower institutional birth rate between 62 and 71%. Four women were selected from urban slums in Patna and four more from rural villages in Muzaffarpur. Women were purposively selected to participate in the research. We went door to door asking whether women reside in each household to recruit women. Eight women who gave birth in the three years before the study, are included as in-depth case studies, and each woman was asked about all of her previous birthing experiences. The eight women present diverse case studies in terms of birth setting, type of birth and number of births, which ensured diversity in the birthing experiences.

The female lead author facilitated the body mapping exercise with women, with assistance from a local female research assistant in Bihar who had experience of qualitative interviewing, and is adept in Bihari dialects and culture. Between two to five individual household visits were undertaken for each participating woman. In some cases, the sessions were conducted before 6 am or after 8 pm to suit women’s convenience. These timings were less disruptive for the interviews. Women were usually busy with their household and outside work during the day and could only give us time after completing the household chores. The first visit involved talking about the research and purpose of the study, seeking consent, reading out the participant information sheet in the local language and dialect, answering initial questions from women and her family before proceeding to start the exercise. Women spent on average 3.7 h in total with us over a week. We carried 3.5 ft by 7 ft sized charts along with artwork essentials and cut-outs that helped to put the story together for co-creating the birthing maps. The final versions of the maps and stories were member checked and approved by the women. Reflexive notes were taken with detailed notes of the interview environment. Each transcript was translated into English by the research assistant. The lead researcher checked each audio recording with the transcript for translation accuracy, to ensure the correctness of the captured narrative.

We faced several challenges during data collection, which sometimes included researchers offering to look after family members, children and pets in the household. Cramped living spaces made it difficult to ensure the privacy of women during data collection, and in some cases there was barely enough room to spread the long sheets. Many women were unable to participate in the exercise because of time constraints. Cooperation of family members was important. We were often required to talk to the mother/ mother-in-laws about the study before we could speak with the women. On a few occasions we were unable to proceed because of objections from family members. Women were sometimes reluctant to participate as most of them did not have the habit of writing and some of them had never held a pen. However, they became more deeply engaged during the process, as they started creating the maps with the researchers. This helped them to share their experiences frankly.

### Data analysis

**Fig. 1:**
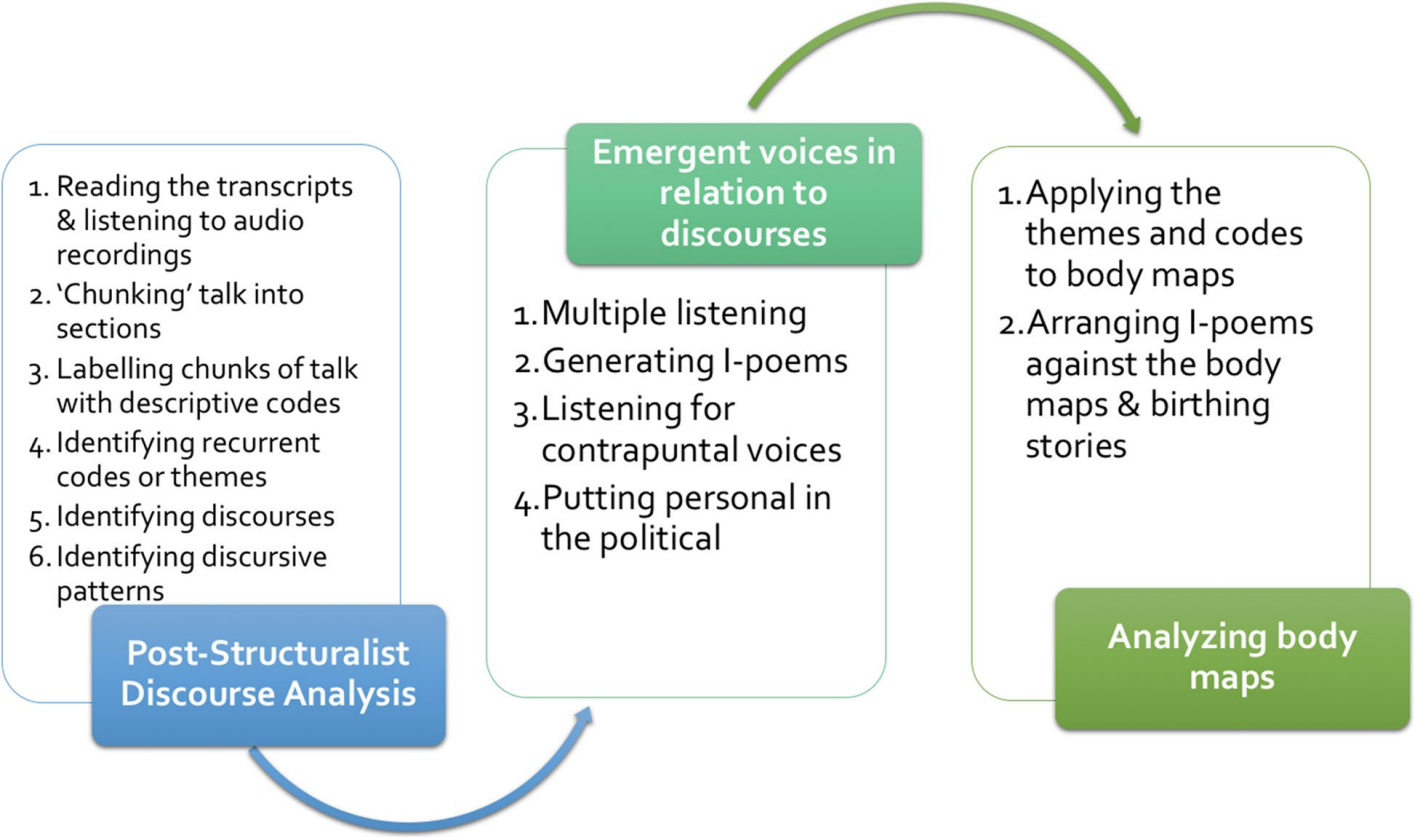
Adapted from feminist relational discourse analysis

Data analysis begun during the fieldwork. Co-creation of the body map and birthing stories could be considered the first round of data analysis. Post fieldwork, the data were analysed using Feminist Relational Discourse Analysis (FRDA) through a ten step process divided into two analytic phases: 1) post-structuralist discourse analysis; 2) emergent voices in relation to discourses (Fig. 1) [39]. Data from each women included the transcript, audio recording, birthing map, birthing story and reflexive notes. It includes creating I-poems, as voice relational discourse analysis is embedded in the analytic process, but they have not been presented in this paper. The third phase of analysis is unique because it involved analysing the birthing maps by applying the themes and codes generated from phase one and two to the maps.

This study required a multilayered analysis, which provided enough opportunities for the women’s voice to take dominance in the analysis over the researcher’s interpretation. The detailed codes included following in-vivo, process, emotion, value, attribute, provisional, causation, simultaneous and sub coding. This was an extensive exercise that took almost 3 weeks to analyse each woman’s data. Analysis was carried out by using a qualitative data analysis software NVivo12.

## Results

The eight women interviewed focused on any one birth experience of their choice in detail to feature on their birthing body map. In total, they had 20 live births (10 each from urban and rural) and two stillbirths; at different levels of government hospital, at private hospitals and at home. Women shared their experience and perceptions of respect, disrespect and abuse during vaginal and cesarean birth. More information about the women is provided in Table 2.

**Table 2:** Participating women’s profile

Pseudonym	Age (Years)	Education	Occupation	Age at childbirth(s) in years	Birth settings
WU01	25	6th standard	Home-maker	19, 21 & 23	Same private hospital for each birth
WU02	32	12th standard	Cleaner & milk seller	26	Birth in tertiary public hospital
WU03	28	12th standard	Home-maker	23, 25, 27 & 28	One birth in a private hospital, the other three in different public hospitals
WU04	19	5th standard	Home-maker	15 & 19	One birth in a private hospital and one home birth
WR05	22	No formal education	Manages a grocery shop	18 & 20	Two births in different primary public hospitals
WR06	22	8th standard	Home-maker	19, 20, 22	Three births in different public hospitals at primary & secondary levels
WR07	25	No formal education	Farm labourer	20, 21, 22, 23 & 25	Births in Govt hospital twice, home birth thrice
WR08	29	MA, B.Ed, BA	Teacher	25 & 28	Births in different private hospitals

Women did not mention words such as “samman” or “izzat” that are used in literary explanations of ‘respect’ and ‘dignity’ in Hindi because the language used by the women was colloquial. They conveyed their feelings through simpler words such as “acchcha” (good) and “bura” (bad). They communicated through facial expressions and by stating whether a particular experience made them feel angry, afraid, shy, ashamed, regretful, let down, or happy. Women described experiences detailing the birth place, interventions, birthing environment, people around birth, being touched during birth and communication. They shared their experiences before they gave birth, how their birthing experiences influenced their subsequent births, and the impact one birthing experience had on another in terms of respect, disrespect and abuse. They explained what they meant by a ‘good birth,’ which also influenced decision making during childbirth, their expectations of future births and births of women around them.

### *‘Good’* birth*, ‘Bad’* birth and expected birth

**Fig. 2:**
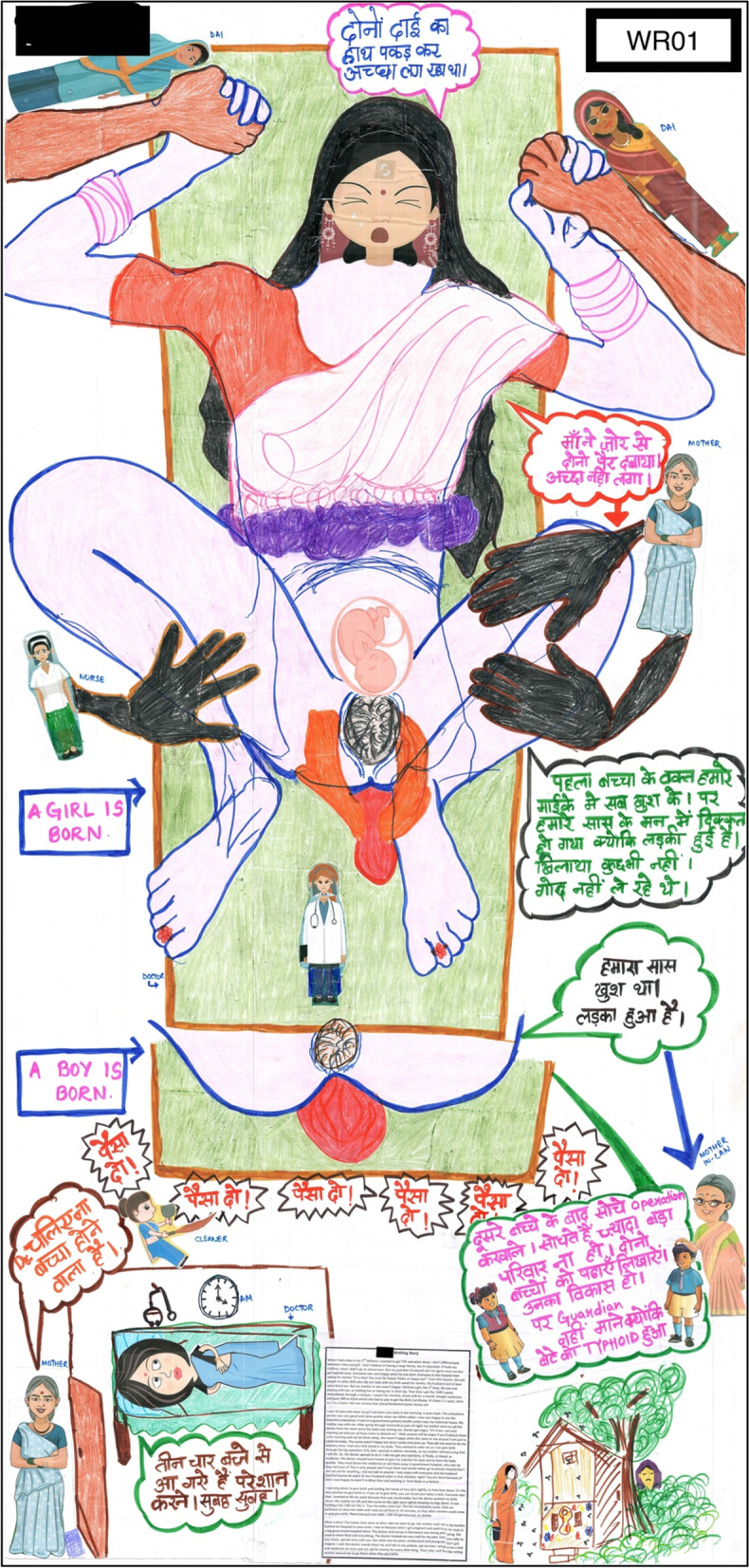
WR05’s birth map of vaginal birth at a primary public health facility

Women were asked to choose one of their birthing experiences, in case of a multigravida, to show on the birthing body map. WR02 was the only primi-gravida participant. WR05, WR08, WU03 and WU01 chose to narrate their most traumatic birthing story on the map. WU04, WR06 and WR07 chose to narrate good birthing experiences. Women were asked to rank their birthing experiences to help them choose which birth to create on the map. This helped women to understand what according to them is a ‘*good birth’* or *‘bad birth’.* A good outcome in their perception was part of a good birthing experience. They reported the birth of a son as a good outcome. This could be an indication of son preference, reflecting the patriarchal societal structure, as can be seen in WR05’s birthing body map (Fig. 2).

*“The boy! I felt better when I had the boy because I had good pain and took very less time. My girl’s birth was very painful for me. I had so much problem. I would like to show the girl’s birth on the map.”* (WR05)

Women’s understanding of an ideal birth was expressed in parts throughout the multiple interactions to the extent where births which were considered *‘better’* became *‘bad’.* As the trust developed over the interactions, they opened up more. The expected birth was asked in terms of respectfulness apart from experienced birth as compiled in Table 3.

**Table 3:** Women’s understanding of good, bad & expected birth experiences

Participating woman (live births)	Experienced birth		Expected respectful birth
	Good Birth	Bad Birth/ Better Birth	
WR05 (2)	-short duration of labour	-too painful -neglect and abandonment -verbal abuse discriminated against on the basis of sex of newborn	-no touching -proper communication -timely examination -no delay in care -polite care provider, serve with a smile -a bed -care provider should treat as family member -non discriminatory care regardless of newborn’s sex -availability of hospital supplies -no disrespect -no extortion -not more than two people around her during childbirth -incentive for institutional birth
WR08 (2)	-better prepared from experience -lower expectation than previous birth	-too many vaginal examinations by different care providers without maintaining privacy or seeking consent - extortion -blindfolded -inhumane treatment -many men around in the birthing room (OT)	-vaginal (normal) birth -comforting touch -no vaginal examinations -seek consent before touching -explanation and consent before interventions -birth companion in OT
WU04 (2)	-home birth -better neonatal outcome -respectful communication in hospital birth	-private hospital birth -unaffordable care -journey to hospital to give birth, travel through a bumpy road -unexplained, unconsented augmentation	-home birth -no vaginal examination
WU02 (3)	-clean toilet -quick ambulance	-swelling of legs and arms -difficulty walking -unconsented and forced vaginal examination -restrained, physical abuse	-no touching -verbal abuse (hoped it won’t happen) -no delay in care -no complications -respectful communication -birth companion of choice -food to be provided at hospital -clean bed, birthing room and bathroom -birth in the hospital - incentive for institutional birth
WU02 (1)	-comforting touch from another birthing woman’s companion -affordable care	-verbal abuse -shouted at -uterine prolapse repair without anesthesia -baby declared dead by *dais* without newborn assessment	-no physical abuse -comforting touch -birthing companion of choice -extortion, but hoped it won’t happen −1:1 care -one bed, one room -curtains for privacy -respectful behaviour from care providers -care providers should introduce themselves -baby should be received with care and assessed properly after birth -proper light & ventilation
WU03 (4)	-presence of ‘*guardian’* though not of choice -care providers followed her believes (norms) after insisting -care providers did not do vaginal examination after refusal	-extreme pain -forgotten to remove gauge piece before episiotomy repair -extortion -unexplained and unconsented vaginal examination, episiotomy, episiotomy repair, uterine exploration and augmentation -no anesthesia before episiotomy repair	-husband’s presence as a birth companion -no episiotomy -birthing woman’s norms and believes to be followed
WU01 (3)	-no medicine -no vaginal examination -less pain -quick delivery -privacy protected	-shouted at, verbally abused -unconsented, unexplained vaginal examination, episiotomy, episiotomy repair, augmentation -restrained, physically abused -detention of newborn -extortion	-no cut -little pain -no stitching without anesthesia -no unnecessary touching -seek permission before touching and interventions -respectful behaviour from care providers -come home alive after birth -no birth companion
WR07 (3)	-home birth -three live births -same *dagarin*	-hospital -two stillbirths	-home births -continuity of care across all births

### Disrespectful and abusive birth

Women referred to various kinds of disrespectful and abusive encounters with the health and non-health care providers during their stay at the hospital. They reported hearing similar experiences from their friends, family members and women in the neighbourhood who often made similar choices of birth place. Stories of respect, disrespect and abuse were kept in mind when giving birth the next time. These stories embodied desired behaviours, self discipline and ways to avoid being mistreated, humiliated and have as close to a dignified birthing experience as possible. Women often said that they discussed mistreatment only in a hushed manner amongst peers when someone is going to give birth. These stories were not shared with authorities as a grievance, not even with senior members of the family including their husband with whom the conversations about birth were rare. All the women had experienced disrespect during childbirth.

Verbal abuse was the most common mistreatment women experienced. Some care provider’s comments were so disgusting that women refused to repeat their words.

*“Two sisters* (nurses) *were very bad because they were abusing me and shouting at me like anything, I can’t even tell you the things they said to me.”* (WU01)

*“The doctor inserted her fingers inside me and I screamed very loudly. She said, ‘WR08 has no pain threshold, she can never have a normal birth!’”* (WR08)

*“She said, ‘Shut up! Why are you screaming so much?’… ‘behave! Look how you are screaming!’”* (WU02)

Incidents of physical abuse were mentioned. Women did not perceive restraining as abuse, but reported that it is common and they considered it as a bad touch but a part of quality care. Women disliked being held down, but were hesitant to say that because often their family members were the ones restraining the women. Episiotomy repair without anaesthesia is a common kind of physical abuse that women (WU02, WU01, WU03) have experienced but they considered that a part of care during childbirth as well. Every woman was touched without consent. They reported feeling *‘uncomfortable’* and *‘ashamed’* but could not say this to the people around them.

*“They do hit women in that condition… A woman had come, she was screaming so much from the pain that she could not stay in her bed. The sister* (nurse) *gave her two tight slaps across her face.”* (WU02)

*“People don’t like being touched but everyone has to go through it!”* (WR06)

Women did not speak about experiencing sexual abuse but the narration and non-verbal communications conveyed otherwise. One of the women referred to comments with sexual connotation as ‘*colourful things’* while also sharing how uncomfortable it made her feel, but she believed that everyone had to experience it.

*“The doctor said ‘you are not scared of other things, of doing it, but you are scared of injections!’ Many people abuse like that. They said ‘if you are so afraid then why did you conceive? When the baby had stayed, you should have taken a medicine to get rid of it! What’s the need of having children then?’”* (WU02)

Extortion is the commonest form of mistreatment that was mentioned by every woman, except WR07. It has become a tradition for care providers to ask for *‘khushi ke paise’* (happiness money) that also determines respectfulness and quality of care based on care seeker’s affordability to tip them. Money is sought as soon as the baby was born. The care providers do not miss an opportunity to demand money, as they do not know when the family might leave the hospital. Women have reported that all types of health (doctors, nurses) and non-healthcare providers (ASHA, Mamta, cleaner, dagarin/ dai) ask for money, however how the money is shared amongst team members is not known. They demand money for everyone in the hospital, regardless of how many people were involved in care provision.

*“Happiness money! She said ‘your grand-daughter is like Goddess Lakshmi’ then everyone asked for money. My husband gave 100-150 to everyone. There were 11-12 people there… Happily!? Some people give it happily but they have made a habit out of it. People who can’t afford, they ask them too. That is greed!”* (WR08)

*“My husband does garbage work* (rag-picker)… *he lost his job and we have 300 rupees. Should we go to hospital or take care of the house! We had no money to go to hospital in the second birth… everyone had taken 100 rupees last time in the name of happiness, there were 9 people.”* (WU04)

A woman respondent mentioned that she had to bribe the Newborn Intensive Care Unit (NICU) staff every time or they didn’t let her visit her daughter. This was traumatic for her. A similar experience was reported by WU01, which led to her baby’s detention in the private hospital until they paid extra over the hospital fees. The baby was kept for hours until the care providers received the *‘happiness money’.*

*“There were two bad sisters* (nurses)*. My father gave them 1000 rupees but they were not happy. They told us that other people give them gold earrings and necklace. My father was not giving them money so they kept the baby for two hours and when my father finally gave more money the sisters* (nurses) *handed over our baby to us.”* (WU01)

*“Some guardians can not pay as much as the doctors ask for. The nurses withhold the baby saying ‘we want this much or we won’t give the baby’. Many people don’t have enough money to free the baby from them. Sometimes it turns into conflicts.”* (WU03)

**Fig. 3:**
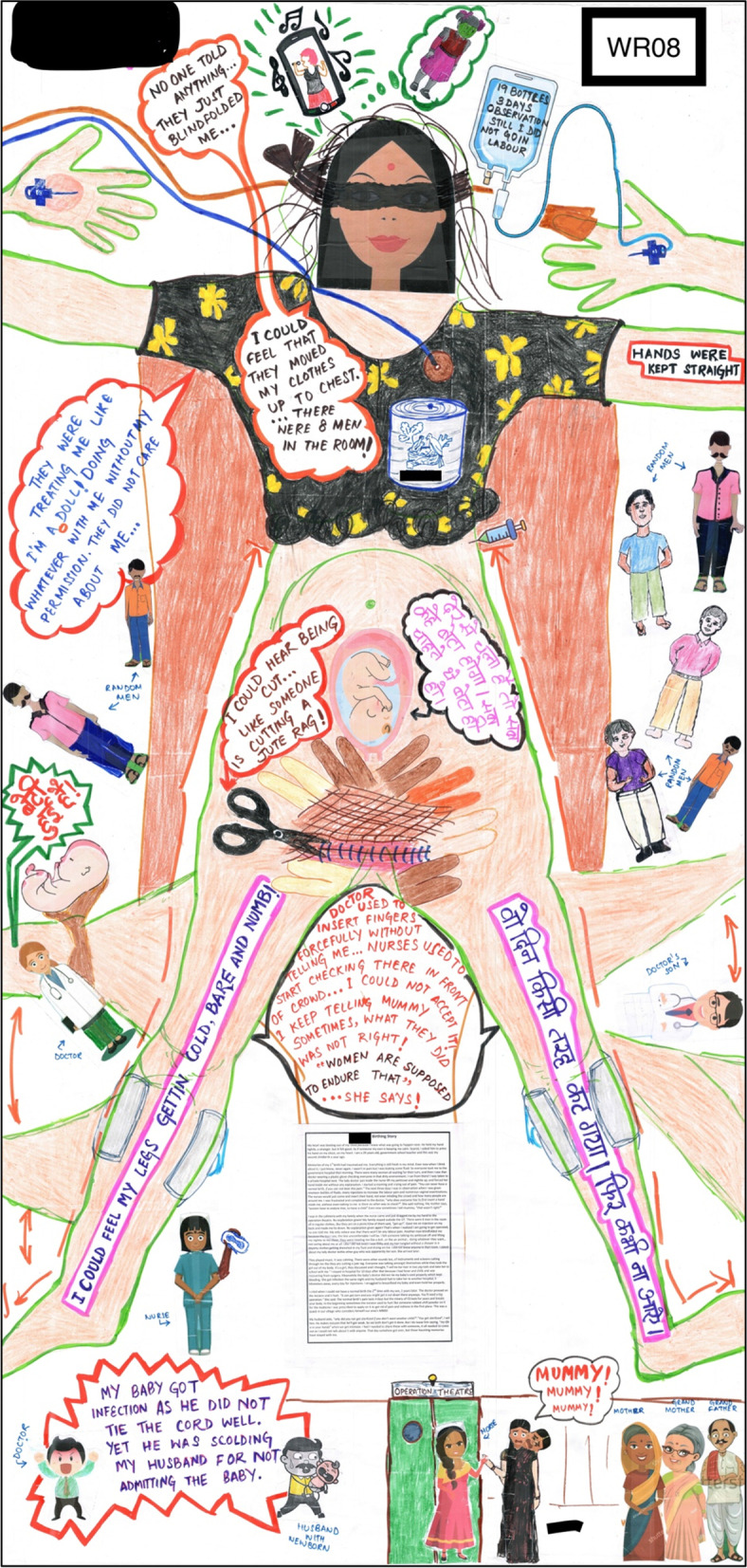
WR08’s birthing map of caesarean birth at a private hospital

Unconsented procedures showed a failure to meet professional standards. There are many instances showing poor rapport between care providers and women. This includes a lack of explanation, detention and treating women as passive objects as shown in WR08’s experience in Fig. 3.

### Respectful birth

**Fig. 4:**
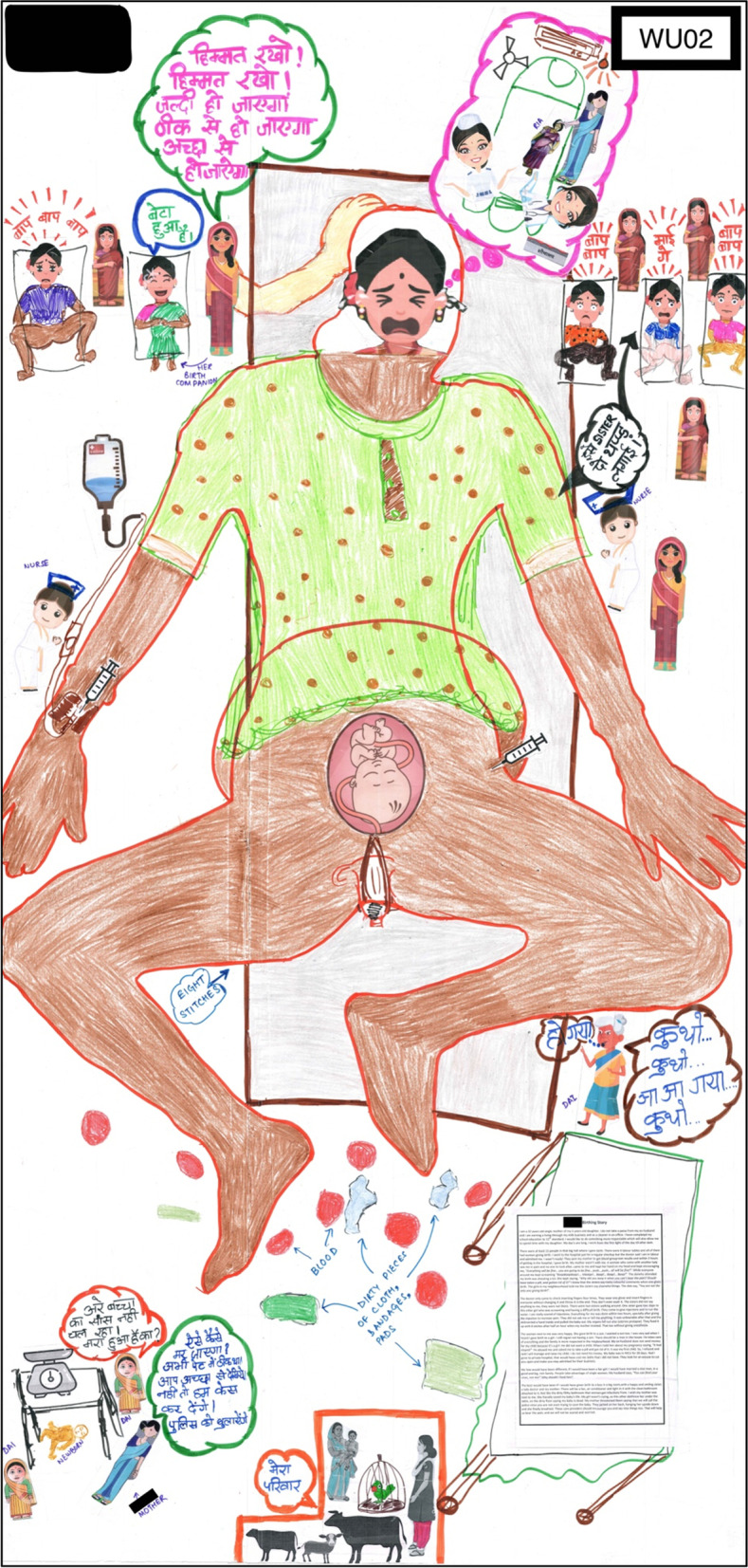
WU02’s birth map of vaginal birth at a tertiary public hospital

Women often just said they felt *‘good*’ when they talked about the respectful aspects of their birthing experience. WR05 had visited a tertiary level hospital in a different state for a few antenatal visits before returning to Bihar to give birth. She talked about the cleanliness and good behaviour of the hospital staff there, while comparing this with her previous birthing experiences. Getting a clean ambulance to travel to the hospital on time, was the only positive aspect about her births (Fig. 4). She wanted cleanliness in the entire hospital. She has a clear vision of what respectful care means to her.

*“Care should be like, when I told them what problem I have, they* (care providers) *should come and check me completely and tell me about my condition, that in how much time I will deliver. I will feel respectful when they will do my delivery on time without delay, when they will speak with me politely with a smile. When they will take care of me nicely. If they gave me a bed… They should treat us like family members. No matter whether I am birthing a boy or a girl, I should be treated well! They should talk to me nicely … no matter whether it is the nurse, doctor or dai. They should give immunisation, injection, medicines and other supplies from hospital, if it is available. If it is not available, they should bring it from outside and give us. Then only we will share our good experience with other women in the neighbourhood, that we are not disrespected there and people are not greedy. What’s the point of going there otherwise!”* (WR05)

A touch that felt good was mentioned as a calming and rarely experienced aspect of birth. WR08 was relieved to have given birth pain-free. After having a traumatic birth experience (Fig. 3), it was difficult for her to go through another cesarean even though she had changed the hospital and the doctor. Memories of her first birth experience caused severe anxiety when she was lying on the operation table, blindfolded, and she got support from a stranger who held her hand and helped her calm down. WU02 has a similar story of her vaginal birth in a public tertiary hospital where she shared the same open space with five other women in labour without any privacy (Fig. 2). Both of them did not have family members present to offer support during childbirth, so strangers gave the support which was the best aspect of their experience. For WR08, it was the *‘stem-cell-guy’* and for WU02 it was the birth companion of another woman birthing next to her. Cord blood banking is available in most private hospitals in India, which explains the presence of this person in the OT whom the WR08 referred to as the ‘*stem-cell-guy’*.

*“There were many people in the OT and I had the stem cell guy in the OT. He held my hand. I knew I was going to be operated so I went completely numb in my heart. It was hurting like an injection due to fear. I told him to press tightly on my heart, on my chest. He offered water but I refused to drink… ‘just press tightly here’ and he did (*laughs*). No one will tell anyone like this* (laughs) *but I was blindfolded and I didn’t even know who he was. I didn’t see his face. After sometime he asked ‘are you okay?’, I said ‘yes’… felt like he is my own, someone familiar.”* (WR08)

Women expect good touch or no touch without consent. WR08 recalled no vaginal examinations being a relief in her second birth after undergoing numerous examinations by different care providers in her first birth (Fig. 3). Respectful care could be seen through usual care giving and unusual ways such as praying.

*“She (*doctor*) was very nice because she also started praying with us* (for the baby). *She was an innocent nice lady. She was so good. I felt good because those were good people, doing good things and taking care of me nicely. We were happy!”* (WU04)

**Fig. 5:**
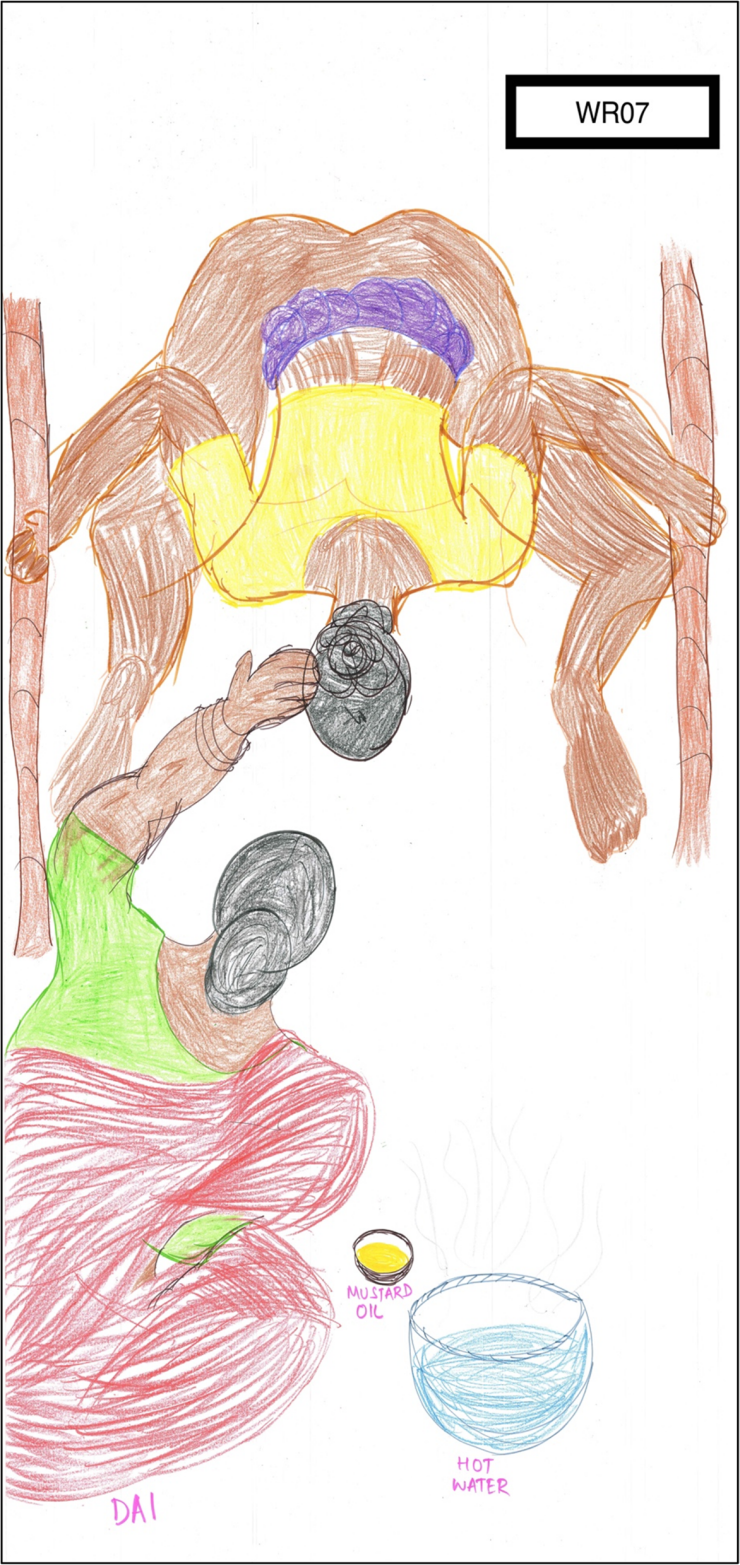
WR07’s birthing map of home birth

Women often changed birth places after a traumatic birthing experience. WR07 gave birth at home thrice after having had two stillbirths at hospitals (Fig. 5). She believes that her babies survived because she gave birth at home. WU03, WR08 and WR06 changed hospitals or birth settings based on their previous experience. WU04 had a hospital birth which wasn’t very bad in her opinion but home birth was affordable.

*“Obviously I will be most respectfully treated at home! You get good services at home. If you give birth at home, then it is home only. There is no need to go anywhere else or reserve a vehicle. There is no need to go running around arranging things. Everything happens at home. The roads have potholes, its bumpy, discomforting while travelling to the hospital. So I gave birth at home.”* (WU04)

Most women who gave birth in a hospital, experienced neglect and abandonment at some point during their stay. Apart from good behaviour, there were birth environment related aspects that should be respectful, in WR06’s opinion.

*“They should talk nicely with everyone. Meals should be given in the hospital. Bathroom should be clean, room should be clean, bed should be clean. If you are alone, you will be scared. No body should touch. I do not want to be touched by anyone. There should be someone with you, it’s important.”* (WR06)

Birth companions are considered important. Birth companions or *‘guardians’* could ensure women are treated respectfully. Their presence means the care providers will receive ‘*happiness money’* after birth, which ensures that they treat women better. A woman’s family is not allowed in the operation theatre, as was seen for WR08. Husbands are usually kept away from birth, and male relatives are not allowed in the birthing room. The issue of male presence also emerges in relation to care providers in the birthing space where the *dagarin*, ASHA, nurses and doctors are usually female but we have seen a doctor and other care providers who were men in WR08’s story, in the operation theatre. One woman wished for her husband’s presence during childbirth but she was aware that it is usually frowned upon and considered shameful, hence she did not make that request to the health care providers. WU04 and WR06 felt there was no need for husband to be there. WR05 blamed her husband for her painful births. WR07’s husband played a key role in all her births as the only person around, other than the *dagarin.* He caught the baby in her first home birth, he waited outside in the two subsequent births. WU01’s husband was in prison one time and chose not to visit her in the next two births. WU02’s husband abandoned her when she conceived. WU03’s husband had to wait outside the birthing room in all four births and she wanted to change that.

*“The benefit would be that when I was crying out of pain, I could have held my husband’s hand. I could have shared my pain with him and that would be good for me (*laughs*). He may not have felt anything, but I would have felt everything. It was all about me, I was in pain so let’s focus on how that would have helped me (*laughs*). I would have benefited from my husband’s presence for sure. He was outside, waiting to hear about the baby. I wanted him in the room with me. If my husband would have been there, I would have asked him to come near me (*laughs*)… But having husband next to you is something different… holding his hand will be more than enough (*laughs*). In this condition, husband is needed more! He can also hug me. I will be in pain, but because of my husband’s company, my pain will be less. I will feel relaxed. Being with him will make me happier.”* (WU03)

Women do not want unnecessary interventions during childbirth. Some of these interventions are perceived as bad and unwelcomed touch. They wanted to give birth with *‘no cuts, no checks’* because episiotomy, repair and vaginal examinations are some of the most disrespectful and abusive accounts narrated by women including unnecessary exposure, that is shameful and traumatic to them. Women wanted privacy to be maintained at all times. Ensuring their norms and beliefs are valued, is important, as WU03 experienced when she felt the baby was not coming out because the fan needed to be switched off to increase her body heat. The nurse did that for her and she felt the baby came out quickly after that. Women want the interventions to be explained to them.

*“They should have explained* (about vaginal examination) *to me. Then I would have thought, okay they will do this to me and it is required. So I would have convinced myself to have courage. I would have prepared myself.”* (WR08)

Many of the women shared what their ‘birthing experience of dreams’ would be like, if they had all the resources imaginable just vested to make their birthing experience more respectful and satisfactory. This is WU02’s hopes for her daughter’s birth (Fig. 2), because she does not plan to give birth again.

*“There should be only one bed, surrounded by curtains on all sides… there should be a sister who will talk nicely and politely with love. They will guide us what to do saying ‘dear this is this and it’s a boy or a girl’… I will give birth lying down only, in a separate room. They should be encouraging. I want to be familiar with them* (doctor, nurse). *I would think that someone is there and I will feel less afraid and not panic. Encouraging environment! Everything should be done with love and care, then I will feel happy and satisfied… once the baby is received with care, check the general condition of the baby, assess the baby thoroughly, identify the presence of any problem… provide care. After cleaning the baby, it should be shown to me so I know whether I birthed a son or daughter. The room should be clean, no smells, the floor should be clean. The bed should be such that the head can be raised up. There should be proper light, ventilation, working air conditioner and running fan specially in hot weather. If television is there that would be better. There should be a bed for my family too in the same room. I should not have to spend so much money.”* (WU02)

Women did not exercise their decision making right in the hospital. Decisions were made by the care providers and sometimes informed to the family, but never consulted with women. Women wanted to be informed about the care they were receiving, involved in decision making, spoken to nicely, and asked if they wanted to receive interventions. However, they were not comfortable expressing this while giving birth in the hospital, unless pushed to extreme circumstances such as repeated vaginal examination without privacy. In addition, women were not comfortable talking about the discomfort and humiliation they experienced and only complained about it when it was intolerably abusive. They felt they were the least of everyone’s concern in the birthing environment.

Women gave birth in a restrained supine position in the hospital and often did not have an opinion on the ideal position of birth. WR07 gave birth squatting in all three of her births at home (Fig. 5), taking support from the two bamboo poles. The same *dagarin* attended all her births which was comforting, as she knew her carer and had developed a relationship of trust. WU04, who gave birth at home with a *dagarin*, felt the presence of her rabbits during her birth as something she would prefer if she gave birth again. WU01 was cared by the same health care team in all the three of her births in a private hospital but continuity of care did not lead to a positive birthing experience for her, instead a pattern of abuse repeated every time and she had no say in choosing where she will give birth.

The final aspect was about the choice of how to give birth and all women wanted to give birth ‘normally’. WR08 spent days crying when she could not give birth vaginally the second time too, because her first birth was cesarean. Women who had a vaginal birth reported having a ‘mini-operation’ (episiotomy), as the most traumatic aspect of their childbirth, as narrated by WU01 and WU02. It was especially traumatic for WU02 (Fig. 2) who suffered from a uterine prolapse followed by a painful repair. Their happiest experiences were that of intervention free vaginal births, but women felt they were deprived of their right to make that choice about their body.

## Discussion

Women were surprised to find someone knocking at their door with the proposition of hearing their birthing stories. Birth is a hidden topic of conversation among women because it is commonly ascribed to as an outcome of sexual intercourse [21, 40]. Women are often verbally abused with judgmental comments using this fact, as is also seen in WU02’s narrative. This is not just about narratives of traumatic birth, the literature suggests that women sharing good birthing experiences are shamed for ‘showing off’ [41]. Given the utility of body maps in understanding women’s experience of crucial life events such as childbirth, our adaptation can be named ‘birthing maps’. The unique application of birthing maps helped to address the language and power barriers and helped women to break their silence especially about their experiences of obstetric violence and share their birthing experience in detail with comfort. It helped to break women’s silence about their birthing experiences, where theirs is the crucial voice driving the improvements in sexual, reproductive and maternal health care [19, 21, 42–44]. Over-medicalisation makes the birth world helpless for women to understand and describe. Studies suggest that the culture of silence and the acclimatisation to tolerate violence in personal lives, often as a result of a patriarchal culture, may keep them from reporting on their experience of disrespect and abuse in the birthing environment, as they may be conditioned to feel less valued [21, 33, 41, 43, 44].

This study is grounded in feminist epistemology and guided by feminist critical theory to understand mistreatment during childbirth. The methods used in the study have ensured that the women’s voice is primary and the author’s interpretation is secondary. This was ensured during data collection, analysis and presentation of the findings by using the birthing body maps, birthing stories and the generous inclusion of quotes.

There were aspects of respectfulness in every woman’s narrative, which were not necessarily received from the care provider, such as the person who comforted WU02 or WR08 in their births in a public and private hospital respectively. The hospital birth setting in a private hospital was not necessarily very different from the public settings at different levels. Women reported that the private hospitals discussed in this study were advantageous when compared with the public hospital in terms of getting a bed or a separate room and receiving quicker attention from the care providers. WU02 and WR08 have birthed in very crowded environments. This is quite the opposite of the comfort drawn from the experience of communal birthing where everyone around the woman is there for her, supporting her and encouraging her [15, 45].

This study highlights the opportunities and scope to ensure respectfulness in birthing experience. In hospital births, respectful care begins with the reception of women. An absence of basic human interaction is the most common form of disrespect that women experience [12]. There is scope for care providers and health systems to embed respectfulness at every point of interaction with women throughout their sexual, reproductive and maternal health needs. Respectful care can be achieved through better interpersonal communication, allowing birth companions of choice, provision of birthing environments that are clean, have access to clean water, and hygiene with proper sanitation. Other aspects of care that need to be in place to ensure respectful care are effective communication, sensitivity, and avoidance of unnecessary interventions, and obtaining informed consent for essential interventions [3, 36].

Medical interventions were prioritised over women’s comfort, dignity and choice [21, 46–48]. Women reported being the last priority in the birthing environment. This is a sad reality that is systemic and a part of the cultural conditioning of women that makes them accept unnecessary interventions including cesarean [47] or as Lambert et al. argues ‘you barter your choice in return for skilled care’ [49]. While literature suggest that “being present with the woman” is crucial for positive birthing experiences [48], in women’s opinion the care providers would rank the foetus / baby and their own convenience as top concerns in the order of preference for care. This is reflected in care providers coercing, compelling and restricting women on choices about their bodies [15, 44]. It is important to understand the difference between having a birth companion chosen by the family and health care providers, and one chosen and preferred by the woman, whose focus is supportive care towards the woman’s needs during the birth process [40]. The continuous presence of a health-care provider with every woman is not reported in these stories from Bihar, apart from a *dagarin* in one case who was with the woman throughout at her home births. Good touch, that is soothing, is highlighted as essential in the good birthing narratives [50] as could be seen in WU03’s expectations. This tactile comfort is usually sought in an atmosphere and relationship that women trust [20, 45]. Our study predominantly reports bad touch, because most of the types of touch women reported during childbirth was uninvited, unconsented and traumatizing. The current global narrative of women’s birth experiences is mostly negative. It includes the exchanges that women have about birth with their female family members or friends. Subsequently, women are preconditioned to expect abusive births unless they behave or abide by the care providers or let them do things and do not come in the way of care and be completely passive, to curtail the harrowing experience.

Women in Bihar request for ‘*dard ki dava’* (augmentation by un-prescribed uterotonics to hasten uterine contractions). They are often augmented by ASHA’s or dais before going to the hospital, to reduce the duration of their stay in the hospital. They do not consider it abuse, as compared to women in high-income countries often do; this has alluded to a better understanding of what entails poor quality and disrespectful care. Thus, there are differences in perceptions of abuse and the extent of acceptability and reporting. This is different in high-income countries because of a better understanding by women of poor quality and disrespectful care. Women’s choices are shaped by previous birthing experience. Thus, women often factor in memories of the respectfulness of the care received, especially when they have previously experienced obstetric violence. More so, women often recover from their previous experience in their subsequent birth by trying to do everything possible to make it a positive experience, as a way to heal from a previous traumatic birth [18, 48, 51, 52]. This was also true for women giving birth for the first time, who modified their behaviour based on what they had heard from women in their neighborhood about childbirth. Women in low and middle-income countries endure and often do not know that care can be better with a positive birth experience, they prepare themselves for a horrifying experience [53]. Women need to be aware that they have a say in what happens to their bodies and how they are treated [23]. This could be attributed to their vulnerability to obstetric violence owing to of the women’s background characteristics such as education, socio-economic status, gender, marital status, religion, age, gravida, caste, class etc. and the difference between her characteristics from that of her care provider [16, 54, 55]. This intersectionality is more commonly explained in terms of race and gender but could be used to understand what drives mistreatment during childbirth [15]. In countries such as India, care providers need to work harder to ensure respectful and person centered care, as they are up against women’s own expectations of being mistreated [53]. Even though some instances of abuse are unintentional or a particular care provider’s fault, they need to be aware that abusive care during birth is the dominant discourse in the Indian birthing culture. Measures are required to address the structural and policy-related drivers that make the women vulnerable to mistreatment during childbirth.

### Strengths and limitations

This study investigated women’s experience of respect, disrespect and abuse during childbirth using a visual arts-based participatory method; body mapping. Adapting this method to birth narratives by producing novel birthing body maps is a key strength of the study. Presenting rich accounts of women’s narratives helps to understand their contextual situations through the maps and their birthing stories, which help the reader to appreciate their perspectives. Women in our study experienced respect, disrespect and abuse in the home environment as well as in private and public facilities, though exploring these perspectives further was beyond the scope of this study. There were aspects of mistreatment or disrespectful behaviour linked to family members or at home that women narrated in regard to birthing experience that had an influence on or added context to their perspectives. Childhood sexual abuse increases women’s vulnerability to abuse during childbirth, but this could not be explored in this study due to added sensitivities to an already sensitive nature of the issue being explored. Women’s experience of respect, disrespect and abuse changes with context and women’s changing perspectives depended on their socio demographic background. It is essential to understand this from the intersectional lens to investigate the role of different cultures, religions, socio-economic background's influence in care around childbirth in order to provide the person-centered care that women deserve.

## Conclusion

Women’s missing voices in research about deeply feminine issues, is a global phenomenon [56]. Because obstetric violence-related research is dominated with surveys and perspectives of others, except the women who give birth. Women remember the trauma of an abusive birth throughout their life, often without an opportunity to share it with anyone. This study makes an important contribution through the birthing maps, a participatory and culturally appropriate visual arts method that is useful for accurately portraying women’s respectful and disrespectful births, and helps women break their silence about obstetric violence and express what they want in terms of respectful maternity care. This is important to understand, as even in the eight case studies we observed diversity in women’s expectations along with some similarities. This diversity can be well understood through birthing maps, as care needs to adapt to these changing circumstances, which is essential to ensure respectful person-centered care during pregnancy, labour and childbirth [11].

The system of care during childbirth is very medicalised in Bihar. The narratives of women from this study and the rampant unethical usage of interventions by care providers, so that they do not have to be at the mercy of the physiological birthing process, perceived as disruptive to their schedule and calendars. WR08’s story clearly shares how “Docsplanation” has taken over the birth world because the medical model has changed to suit the needs of obstetricians, especially male [57]. Remnants of this can be seen in hospital birthing environments in India where women are restrained when giving birth. The presence of eight men in the OT for WR08, none of whom she had met before, is a severe violation of her privacy but instead of keeping the comfort in mind, she was non-consensually blindfolded, which could signify a way of silencing women. All women have a universal right to respectful maternity care, regardless of the context and diverse backgrounds. These rights are important to be ensured in the face of a global pandemic, when ensuring positive birthing experience has been a greater challenge in an atmosphere of fear. Care seeking and care provision has been challenging while segregating COVID-19 positive women from COVID-19 negative women. Resulting in barriers to birth companionship, longer waiting times and denial of services due to limited understanding of the impact of COVID-19 on pregnant women and the denial of vaccination to the increase in abusive care as a case where a care provider threatened to slap the woman in labour if she removed her mask [58–61]. This makes it important and more urgent to understand women’s choices, experiences, and expectations through innovative methods like birth mapping to inform changes in practices and policies around childbirth, which are diverse across cultures and contexts.

## Supplementary Information

**Additional file 1.**

## Data Availability

The information generated from the eight women in this study have been fully analysed and are available from the corresponding author on reasonable request.

## Abbreviations

FRDA: Feminist Relational Discourse Analysis
JSY: Janani Suraksha Yojana
NFHS: National Family Health Survey
QOC: Quality of Care
WHO: World Health Organisation
