# Supplementary material for: Breaking the silence about obstetric violence: Body mapping women’s narratives of respect, disrespect and abuse during childbirth in Bihar, India
Source: BMC Pregnancy Childbirth. 2022 Apr 14;22:318. doi: 10.1186/s12884-022-04503-7 (PMC9009281; doi:10.1186/s12884-022-04503-7)
Supplement: Supplementary file 1 — Additional file 1. [file 12884_2022_4503_MOESM1_ESM.docx]

**Appendix 1**

| **WR05’s Birthing Story**  When I had a boy in my 2nd delivery I wanted to get THE operation done. I don’t differentiate between a boy and girl. I don’t believe in having a large family, but I want to educate both my children, since I didn’t go to school ever. But my guardian (husband) did not agree since my boy got typhoid once. Everyone was very happy when he was born. Everyone in the hospital kept asking for money “It’s a boy! You must be happy! Make us happy too!”. Even the cleaner, dai and people in other shift who did not help with my birth asked for money. My husband and in-laws were there too. But my mother in law wasn’t happy I birthed a girl, the 1st time. No one was playing with her, or holding her or taking her in their lap. That time I got the 1400 rupees immediately through a cheque. I used it for Horlicks, drank milk for a month, bought medicines and gave 300 to ASHA which she had to pay to get the Birth Certificate. It’s been 1.5 years since my boy was born, I did not receive that (Janani Suraksha Yojana) money yet.  I was 18 years old when my girl was born very early in the morning, 4 years back. The ambulance service was very good and came quickly when our ASHA called. I was very happy to see the beautiful ambulance. It was in a government primary health centre near my mother’s house. My mother was with me. After going through tremendous pain all night my mother went to call the doctor from her room since the baby was coming out. Doctor got angry, “it’s 4 am, not even morning yet and you all have come to disturb me”. Well, anyone will be angry if you’ll disturb them early morning and not let them sleep. She wasn’t happy when she came to me at around 5 am just to catch the baby. The nurse wasn’t happy too, since I woke everyone up. They did not want to do my delivery since I had very little blood in my body. They wanted to refer me so I can give birth through the big operation (CS). But I wanted to deliver ‘normally’, so my mother started crying thinking I will die in the process. So, the doctor agreed to do it. I did not get any injections, or fluids, or blood, or medicine. The doctor should have known to give me injection for pain and to have the baby quickly. They must throw the medicines or sell them away in government hospital, why else do they not have it? This is why people don’t trust them and would rather go to private hospital. But I did not ask for anything, I did not talk to anyone. I was angry with everyone and my husband. You’ll of course be angry at your husband when in that situation, right? You are there because of him! I was happy he wasn’t in Bihar then and was working in Tamil Nadu, in a factory.  I was lying down to give birth and holding the hands of two dai’s tightly, to help me bear down. It’s the best position to give birth in. If you sit to give birth, you can break your baby’s neck. Everyone says that. I wanted to lift my waist because that was comfortable, but the doctor pushed my body down. My mother on left and the nurse on the right were tightly keeping my legs down. It was hurting a lot, I did not like it. Then the baby came out. The dai immediately came, took my petticoat to clean the table and I was out of there in 10 minutes, so that other women could come in and give birth. There was just one table. I did not get any cuts or stiches.  Bihar is dirty! The toilets there were so dirty, I did not want to go. My mother took me in the bushes behind the hospital to pass urine. I was in Haryana when I got pregnant and used to go for tests in a big government hospital there. The doctor and nurses in there were very loving and caring. We used to share food and everything. The doctor treated me very well for my pain. Everyone talks to you nicely, spends time with you. But when you are poor, uneducated and young you don’t get respect. I wish the doctor would check me and talk to me politely, ask me how I am, give me a bed and medicines on time and not ask for money for every little thing. Then only I will feel like telling women around me to go there when they give birth. |
| --- |

**Appendix 2**

| **WR08’s Birthing Story**  My heart was beating out of my chest, because I knew what was going to happen next. He held my hand tightly, a stranger, but it felt good. As if someone my own is keeping me calm. Scared, I asked him to press his hand on my chest, on my heart. I am a 29 years old, government school teacher and this was my second childbirth a year ago.  Memories of my 1^st^ birth had traumatized me. Everything is still fresh in my mind. Even now when I think about it, I just know, never again. I wasn’t in pain but I was leaking some fluid. So everyone took me to the government hospital that morning. There were many women all waiting for their turn, and then I saw that doctor wearing a plastic glove checking everyone in that dirty environment. I ran from there! I was taken to a private hospital next. The lady doctor just made the nurse lift my petticoat and nightie up; and forced her hand inside me without any explanation. I started screaming and crying out of pain. “*You can never have a normal birth, if you can not bear this pain.”* The next three days I was in observation when I was given nineteen bottles of fluids, many injections to increase the labour pain and numerous vaginal examinations. The nurses would just come and insert their hand, not even minding the crowd and how many people are around me. I was frustrated and complained to the doctor, “*why does everyone has to first insert a hand inside me, without even talking to me. Is there no other way to check?”.* She said nothing. My mother says, *“women have to endure that, to have a child”.* Even now sometimes I tell mummy, “*that wasn’t right!”*  I was in the cafeteria with my family when the nurse came and just dragged me by my hand to the operation theatre. No explanation given! My family stayed outside the OT. There were 8 men in the room all in regular clothes, like they are on a picnic! One of them said, *“get up!”.* Gave me an injection on my back and made me lie down. No explanation given again! That’s when I realized I am going to get operated, no one told me. My only solace was that there won’t be any labour pain. Another man blindfolded me because the less I see, the less uncomfortable I will be. I felt someone talking my petticoat off and lifting my nightie to my chest. They were treating me like a doll… or like an animal… doing whatever they want… not caring about me at all. Like I did not exist! I was filthy and my hair tangled without a shower in 4 days my clothes getting drenched in my fluid and drying on me. I did not know anyone in that room. I asked about my lady doctor to this other guy who was apparently her son. She arrived later.  They played music. It was calming. There were other sounds too, of instruments and scissors cutting through me like they are cutting a jute rag. Everyone was talking amongst themselves while they took the girl out of my body. It’s a girl, they discussed and I thought, *“I will tie her hair in two pig-tails and take her to school with me.”* I stayed in hospital for 10 days after that because I had fever and chills and was recovering from surgery. Meanwhile the baby’s doctor did not tie my baby’s cord properly which kept bleeding. She got infection the same night and my husband had to take her to another hospital, 3 kilometers away, every day for injections. I struggled to breastfeed my baby and even hold her properly.  I cried when I could not have a normal birth the 2^nd^ time with my son, 2 years later. The doctor pressed on the incision and it hurt. *“it can get torn and you might get a cut down there anyways. You’ll need a big operation.”* She said. The normal birth’s pain lasts 4 days but the misery of CS lasts for years and breaks your body. In the beginning sometimes the incision used to hurt like someone rubbed *chilli* powder on it for the medicine I was prescribed to apply on it to get rid of pain and redness in the first place. This was a quack in our village who considers himself our area’s MBBS!  My husband asks, “*why did you not get sterilized if you don’t want another child?”.* “*You get sterilized”,* I tell him. He makes excuses that he’ll get weak. So we both don’t get it done. But I do tease him saying, “*my life is in your hands”* when we get intimate. I feel I needed to share these with someone, it all needed to come out as I could not talk about it with anyone. That day somehow got over, but those haunting memories have stayed with me. |
| --- |

**Appendix 3**

| **WU02’s Birthing Story**  I am a 32 years old single mother of my 5 years old daughter. I do not take a paisa from my ex-husband and I am earning a living through my milk business and as a cleaner in an office. I have completed my school education to 12th standard. I would like to do something more respectable which will also allow me to spend time with my daughter. My day’s are long, I work from the first light of the day till after dark.  There were at least 15 people in that big hall where I gave birth. There were 6 labour tables and all of them had women giving birth. I went to the hospital just for a regular checkup but the doctor said I am in labour and admitted me. I wasn’t ready! They sent my mother to get blood group test results and within 3 hours of getting in the hospital, I gave birth. My mother wasn’t with me. A woman who came with another lady saw me in pain and no one to look after, came to me and kept her hand on my head and kept encouraging me, “Everything will be fine… you are going to be fine… push… push… all will be fine!”. While everyone around me kept screaming “Baap Baap Baap!…. Maai ge!… Baap!… Baap!… Baap!”. The dai who attended my birth was shouting a lot. She kept saying, “Why did you keep it when you can’t bear the pain? Should have taken a pill, and gotten rid of it!” I knew that the sisters say nasty colourful things when one gives birth. The girls in my neighbourhood told me the sisters say shameful things. The dai says, “You are not the only one giving birth!”.  The doctor only came to check by inserting fingers four times. They wear one glove and insert fingers in everyone without changing it and throw it in the end. They don’t even wash it. The sisters did not say anything to me, they were not there. There were two sisters walking around. One sister gave two slaps to this other girl who was screaming and having a difficult birth. They come to give injections and to run the water. I am really scared of injections. Everything for me was done within two hours, specially after giving the injection to increase pain. They did not ask me or tell me anything. It was unbearable after that and the dai inserted a hand inside and pulled the baby out. My body parts fell out also (uterine prolapse). They fixed it up with 8 stiches after half an hour when my mother insisted. That too without giving anesthesia.  The woman next to me was very happy. She gave birth to a son. I wanted a son too. I was very sad when I heard I gave birth to a girl. I still regret not having a son. There should be a man in the house. He takes care of everything and the family is more respected in the neigbourhood. My ex-husband does not send money for my child because it’s a girl. He did not want a child. When I told him about my pregnancy saying, “It had stayed!” He abused me and asked me to take a pill and get rid of it. It was my first child. So, I refused and said I will manage and raise my child. I do not need his money. My baby was in NICU for 20 days. Had I gone to private hospital, that would have cost me lakhs that I did not have. They look for an excuse to cut you open and make you stay admitted for their business.  My fate would have been different, if I would have been a fair girl. I would have married a nice man, in a good and big, rich family. People take advantage of single women. My husband says, “You can feed your cows, not me!”. Why should I feed him?  The best would have been if I would have given birth to a boy in a big room, with a happy and smiling sister; a lady doctor and my mother. There will be a fan, air conditioner and light in it with the clean bathroom attached to it. Not like the dirty filthy bathroom that women get infections from. I wish my mother was next to me. She literally saved my baby’s life. My girl wasn’t crying, so this other dai threw her under the table, on the dirty floor saying my baby is dead. My mother threatened them saying that we will call the police since you are not even trying to save the baby. They patted on her back, hanging her upside down and she finally breathed. These care providers should encourage you and say nice things too. That will help us bear the pain; and we will not be scared and worried. |
| --- |

**Appendix 4**

| **WR07’s Birthing Story**  I can’t remember at what age I got married. I got pregnant immediately after. I am 25 years old now. I have been pregnant every year since the last five years. I am not educated. I work in other’s farm as and when I get work. My husband does not work anymore since he has been suffering from TB. I manage all the household expenses with around 1000 rupees that I earn every month.  My babies died in hospital twice. So I gave birth to all my three children I conceived next at home with a *dagarin* (Dai). My eldest is a girl and the younger two are boys. The baby used to be fine and I had to take injections every month all through my pregnancy in all the 5 pregnancies in the hospital. But my babies did not service the 1^st^ two times. They would kill my baby every time. Then the doctor used to do D and C (Dilatation and Curettage) to take the dead baby out. Every time it used to stay alive in my womb for 9 months and then die when I went to hospital. So I had all my babies at home and they have all survived. Even in the last three pregnancies I went to hospital for the injections to help continue my pregnancy, get iron tablets and tetanus toxoid injections but I did not go there for delivery. I can’t have children anymore. I got the big operation (tubectomy) done. They gave me 2000 rupees, but there is a lot of pain at the incision. I can’t walk much, or move or bend. Near our village there was this woman whose baby got poisoned inside the belly because it was dead inside her women for 3 months and was rotting. The baby died and soon after the woman died as well. In my case I survived even though my baby got poisoned inside the womb.  This is my mother’s house. I won’t get rest at my in-laws when I get the operation done. So, I came here to have the surgery and recover. I don’t have water supply or electricity at home and I am the sole earning member since my husband is suffering from tuberculosis. But due to my surgery, no one is earning and my mother gives some money to feed us.  At home every time I got pain my husband used to go bring the dagarin (Dai) for delivery. He used to wait outside while the dai helped me deliver inside the room. I would sit and deliver every time holding the two bamboo poles for support to help me bear down. The dai used to have blade, thread, Dettol and hot water with her. She used to cut the cord with blade and use mustard oil to massage my hands and legs when I had contractions. Even after I gave birth she massaged my body with mustard oil and also massaged my baby. It used to be just me and my dagarin in the room all night.  I have two boys and a girl. My youngest boy is 2 months old. All my births have happened at home. Not here, at my *sasuraal* (in-laws). My first two babies broke at the government hospital, they were poisoned in the womb. I had gone to get checked the 1^st^ time when I had pains but the nurse checked me and said I wont give birth now. Then two months later my husband called the ambulance when I got pains but my baby broke. The doctor did D and C to take it out. The second time also the same thing happened. Everytime they used to kill my baby, so the third time I fell pregnant I decided to give birth at home. But I used to get treatment from a private doctor who used to give me two injections every month to help me retain the baby. I used to get bleeding throughout my pregnancy. That happened every time. But everytime I have given birth at home the baby has lived. As soon as I get pain, my husband used to go call the *dagarin* and the 1^st^ time the baby came out before the *dagarin* arrived. Only my husband is there to help me and the *dagarin* does everything from massaging me and the baby, cutting the cord with a blade. I have two bamboo pole to hold when I bear down with the support of that. |
| --- |
